# Differential contractile response of critically ill patients to neuromuscular electrical stimulation

**DOI:** 10.1186/s13054-019-2540-4

**Published:** 2019-09-10

**Authors:** Julius J. Grunow, Moritz Goll, Niklas M. Carbon, Max E. Liebl, Steffen Weber-Carstens, Tobias Wollersheim

**Affiliations:** 1 Department of Anesthesiology and Operative Intensive Care Medicine (CCM, CVK), Charité – Universitätsmedizin Berlin, Corporate Member of Freie Universität Berlin, Humboldt Universität zu Berlin and Berlin Institute of Health, Augustenburger Platz 1, 13353 Berlin, Germany; 2 Department of Physical Medicine and Rehabilitation, Charité – Universitätsmedizin Berlin, Corporate Member of Freie Universität Berlin, Humboldt Universität zu Berlin and Berlin Institute of Health, Charitéplatz 1, 10117 Berlin, Germany; 3 Berlin Institute of Health, Berlin (BIH), Anna-Louisa-Karsch-Str. 2, 10178 Berlin, Germany

**Keywords:** Neuromuscular electrical stimulation, Intensive care unit-acquired weakness, Critical illness, Critical illness myopathy, Early mobilisation

## Abstract

**Background:**

Neuromuscular electrical stimulation (NMES) has been investigated as a preventative measure for intensive care unit-acquired weakness. Trial results remain contradictory and therefore inconclusive. As it has been shown that NMES does not necessarily lead to a contractile response, our aim was to characterise the response of critically ill patients to NMES and investigate potential outcome benefits of an adequate contractile response.

**Methods:**

This is a sub-analysis of a randomised controlled trial investigating early muscle activating measures together with protocol-based physiotherapy in patients with a SOFA score ≥ 9 within the first 72 h after admission. Included patients received protocol-based physiotherapy twice daily for 20 min and NMES once daily for 20 min, bilaterally on eight muscle groups. Electrical current was increased up to 70 mA or until a contraction was detected visually or on palpation. Muscle strength was measured by a blinded assessor at the first adequate awakening and ICU discharge.

**Results:**

One thousand eight hundred twenty-four neuromuscular electrical stimulations in 21 patients starting on day 3.0 (2.0/6.0) after ICU admission were included in this sub-analysis. Contractile response decreased from 64.4% on day 1 to 25.0% on day 7 with a significantly lower response rate in the lower extremities and proximal muscle groups. The electrical current required to elicit a contraction did not change over time (day 1, 50.2 [31.3/58.8] mA; day 7, 45.3 [38.0/57.5] mA). The electrical current necessary for a contractile response was higher in the lower extremities. At the first awakening, patients presented with significant weakness (3.2 [2.5/3.8] MRC score). When dividing the cohort into responders and non-responders (> 50% vs. ≤ 50% contractile response), we observed a significantly higher SOFA score in non-responders. The electrical current necessary for a muscle contraction in responders was significantly lower (38.0 [32.8/42.9] vs. 54.7 [51.3/56.0] mA, *p* < 0.001). Muscle strength showed higher values in the upper extremities of responders at ICU discharge (4.4 [4.1/4.6] vs. 3.3 [2.8/3.8] MRC score, *p* = 0.036).

**Conclusion:**

Patients show a differential contractile response to NMES, which appears to be dependent on the severity of illness and also relevant for potential outcome benefits.

**Trial registration:**

ISRCTN ISRCTN19392591, registered 17 February 2011

**Electronic supplementary material:**

The online version of this article (10.1186/s13054-019-2540-4) contains supplementary material, which is available to authorized users.

## Introduction

Critical illness and intensive care treatment can lead to severe impairments of physical function, cognitive capabilities and mental health, which directly affect the individuals’ daily living, e.g. return to work [1–8]. The physical impairments, in terms of muscular weakness and reduced walking distance, directly affect short-term outcome, as patients suffering from weakness are less likely to be weaned from mechanical ventilation, discharged from the hospital or survive [4]. These impairments can persist at least up to 5 years after discharge from the intensive care unit (ICU) [9]. Therapeutic options that have been the focus of investigations throughout the last decade are early mobilisation and neuromuscular electrical stimulation (NMES). Both of these options have yielded positive as well as negative results in recent randomized controlled trials [10–14]. NMES is able to elicit muscle contractions in unconscious or sedated and therefore uncooperative patients. As it is possible to initiate this form of treatment in the early phase of critical illness, even if the patient has to remain heavily sedated, it was reasoned to be a promising therapeutic option to target muscular pathophysiological processes in their beginning stages [15, 16]. While some trials were able to corroborate this hypothesis, others yielded negative results [13, 14, 17]. Fossat and colleagues found no improvement in muscle strength or mobility in 314 patients receiving NMES of the M. quadriceps femoris in conjunction with in-bed-cycling [13]. An underrecognised aspect throughout recent NMES trials is the fact that not every electrical impulse necessarily elicits an appropriate muscle contraction, as Segers et al. showed [18]. Sepsis, use of vasopressors and lower limb edema were independent predictors for a missing contractile response to NMES [18]. It remains unclear whether neuromuscular electrical stimulation per se has a beneficial effect on the patient or if the beneficial effect is a causal effect of the evoked muscle contraction, which would be in line with current knowledge about muscle physiology. In this case, the lack of contraction from the electrical stimulation would leave study results prone to bias. Furthermore, it remains unclear whether the electrical stimulus during NMES has harmful effects when not properly translated into a muscle contraction. Our aim was, therefore, to characterise the response of critically ill patients to NMES, determine predictors for an adequate or inadequate therapeutic response and investigate potential benefits or detriments of an adequate respectively inadequate muscular response to NMES.

## Methods

### Study design

This is a prospectively planned sub-analysis of a randomised controlled exploratory interventional trial (ISRCTN19392591) performed in two ICUs at the Charité – Universitätsmedizin Berlin, a tertiary referral centre. Ethical approval was granted by the institutional review board (Charité EA 2/041/10).

### Patients

Mechanically ventilated patients ≥ 18 years of age were included on the basis of a Sepsis-related Organ Failure Assessment (SOFA) score ≥ 9 within the first 72 h after ICU admission as well as written informed consent by a legal proxy and excluded if any of the following applied: prior hospital treatment for longer than 7 days, illness prohibiting early mobilisation, pre-existing neuromuscular disease, insulin-dependent diabetes mellitus, Body Mass Index > 35 kg/m^2^, not ambulating before admission or poor prognosis with a high likelihood of death within the next hours. Only patients randomised to receive NMES were considered in this sub-analysis (for detailed study enrolment criteria, see [19]). Study staff had no influence on treatment decisions and patients were treated according to published standard operating procedures [20]. In order to investigate if an adequate response to the NMES had an influence on outcome and to define predictors for an adequate response, we separated the population into responders, defined as > 50% contractile response to NMES, and non-responders, with ≤ 50% contractile response to NMES during the first 7 days of the study intervention.

### Intervention

NMES was applied daily, beginning on the day of enrolment up to day 28, for 20 min per muscle group bilaterally on eight different muscle groups (M. tibialis anterior, M. triceps surae, M. vastus lateralis, posterior thigh, M. biceps brachii, M. triceps brachii, wrist extensors, wrist flexors). We utilised two devices of whom each could stimulate four muscle groups simultaneously. We never stimulated counteracting muscle groups at the same time. Electrical impulses of 350 μs duration were applied at a frequency of 50 Hz. On-time was 6/10 s and off-time was 10/15 s with a ramp of 1 s (MUSKELaktiv 2-Kanal, schwa-medico®, Germany/ Physiomed-Expert-2-Kanal, Physiomed®, Germany). Electrical current was increased until muscle contraction was visible or palpable and then maintained at this level for the remainder of the session. In case pain threshold was reached in awake patients, electrical current was reduced to the last level below the pain threshold and stimulation was performed at that level. If a current of 70 mA failed to evoke a sufficient response, electrical current was not increased further, and the stimulation level was set to 40 mA for the session (Additional file 1: Table S2a). Data on contraction as well as electrical current were documented separately for every NMES session as well as muscle stimulated. For our analysis, only electrical currents from stimulations that lead to a contractile response were respected.

### Outcome

Muscle strength was assessed via Medical Research Council (MRC) score on the first day patients were sufficiently awake as well as at discharge from the ICU. Only patients with an MRC assessment at both timepoints were included in MRC analysis. Mean MRC scores were calculated for each patient. We excluded specific muscle groups from mean MRC score calculation if valid muscle strength assessment was not feasible due to clinical reasons (e.g. external fixators or casts). The day patients were sufficiently awake was determined by fulfilment of the following criteria on two consecutive days: first, Richmond Agitation and Sedation Score between − 1 and + 1, and second, an adequate response to three or more out of the following five verbal commands: “Open/close your eyes,” “Look at me,” “Open your mouth and put out your tongue,” “Nod your head” and “Raise your eyebrows when I have counted up to 5.” Contractile response to the electrical muscle stimulation was confirmed if a muscle contraction was either visible or palpable, or in the case patients were sufficiently awake, if a contraction was reported.

Non-excitable muscle membrane as an electrophysiological predictor for Critical Illness Myopathy was diagnosed via a direct muscle stimulation compound muscle action potential cut-off value of < 3.0 mV [21].

We included the first 7 days of the ICU stay into our investigations as it has been shown that pathophysiological processes already enormously influence the patient during that time and our intervention, specifically NMES, is predestined for the early phase of the disease trajectory as it can be properly applied in sedated, unconscious and uncooperative patients.

### Statistical analysis

Values for categorical variables are shown as count and percentage, while values for metric variables are shown as median and interquartile range (IQR). Statistical difference for metric variables was tested with the Mann-Whitney *U* test for independent samples and Wilcoxon test for dependent samples. Chi-square test was used to test for statistical difference for categorical variables. A receiver operating characteristics (ROC) analysis was performed to determine a SOFA score cut-off value that predicts non-responders to NMES as well as a mA value that is sufficient to stimulate all responders. Sensitivity, specificity and area under the curve (AUC) are reported for the receiver operating characteristics analysis. The Pearson correlation coefficient (*r*^2^) was calculated to determine correlations between variables. A priori, a *p* value < 0.05 was defined to indicate statistical significance.

## Results

During the 2-year inclusion period, 468 out of 3147 patients that were admitted to two ICUs within the Charité – Universitätsmedizin Berlin fulfilled the inclusion criteria. Fifty of these patients were successfully enrolled in the trial and randomised. Further information regarding the enrolment process has been published before [19]. For this sub-analysis, only patients receiving NMES (*n* = 21) were considered (Table 1).

**Table 1:** Baseline characteristics

*n*		21
Sex (m/f)		16/76.2% / 5/23.8%
Age (years)		53.0 (45.0/70.0)
Weight (kg)		85.0 (75.0/100.0)
Height (m)		1.78 (1.74/1.80)
BMI (kg/m^2^)		27.1 (24.2/30.9)
Diagnosis responsible for ICU admission	ARDS	8/38.1%
	Sepsis	3/14.3%
	Multiple trauma	7/33.3%
	Neurologic	2/9.5%
	Miscellaneous	1/4.8%
SOFA at ICU admission		13.0 (11.0/15.0)
APACHE II at ICU admission		25.0 (20.0/28.0)
SAPS2 at ICU admission		58.0 (47.0/65.0)
GCS at ICU admission		5.0 (3.0/6.0)
Time until first awakening (days)		17.0 (10.0/25.0)
ICU length of stay (days)		32.0 (21.0/43.0)
Percent of days with RASS > − 3 during ICU stay		64.3 (37.5/79.3)
Noradrenalin (μg/kg min)		0.07 (0.05/0.11)
Time requiring noradrenalin (days)		12.0 (9.0/18.0)
Survivors/non-survivors		18/85.7% / 3/14.3%
Non-excitable muscle membrane/excitable muscle membrane		7/50% / 7/50%
Start of NMES treatment after ICU admission (days)		3.0 (2.0/6.0)

Values for metric variables are presented as median and interquartile range and for categorical variables as count and percentages. BMI = Body Mass Index; SOFA = Sepsis-related Organ Failure Assessment; ICU = intensive care unit; APACHE II = Acute Physiology and Chronic Health Evaluation II; SAPS 2 = Simplified Acute Physiology Score 2; GCS = Glasgow Coma Scale; RASS = Richmond Aggitation Sedation Scale; NMES = Neurmuscular electrical stimulation

**Fig. 1:**
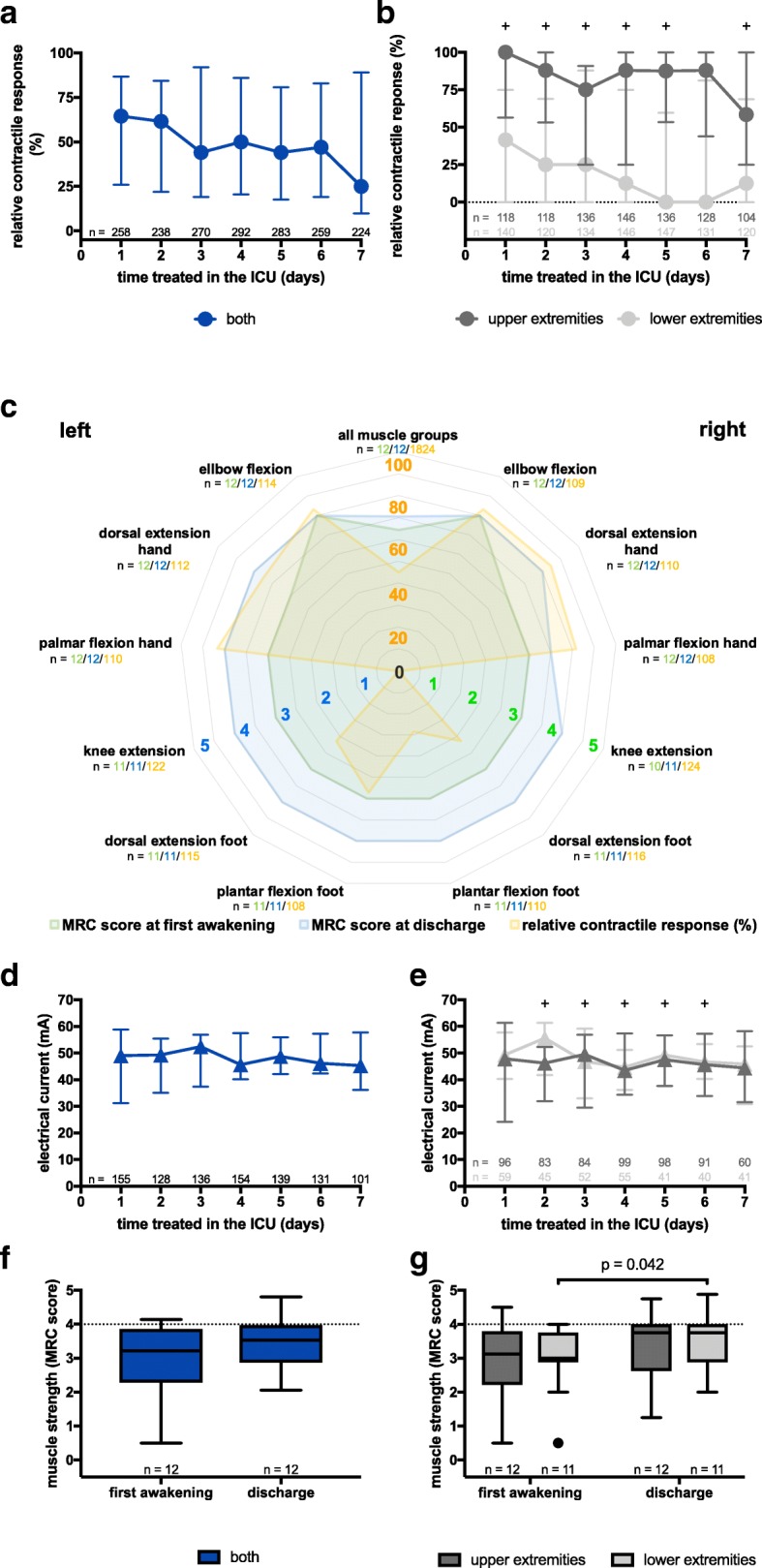
Relative contractile response, electrical current and muscle strength during NMES. **a** Relative contractile response decreases between day 1 and day 7 without reaching statistical significance. **b** Upper extremities show a significantly higher response rate to NMES on days 1, 2, 3, 4, 5 and 7 in comparison to lower extremities. **c** Muscle strength and contractile response for all muscle groups separately. M. vastus lateralis shows the lowest response to NMES. **d** Electrical current required to elicit a muscle contraction remains unchanged between day 1 and day 7. **e** A significant difference in electrical current required to elicit a muscle contraction can be observed on days 2, 3, 4, 5 and 6 when comparing upper and lower extremities. **f** Muscle strength increase for both extremities between first awakening and ICU discharge does not reach statistical significance. **g** The increase in muscle strength between first awakening and ICU discharge reaches statistical significance for lower extremities. All values are shown as median and interquartile range. Statistical significance was calculated via Mann-Whitney *U* test or Wilcoxon signed-rank test as appropriate. A *p* < 0.05 is indicated by "+" in **b** and **e**. ICU = intensive care unit; MRC = Medical research council

In the 21 patients treated with NMES, a total of 1824 muscle groups were stimulated during the first 7 days of treatment. These stimulations were equally distributed between upper (*n* = 886) and lower (*n* = 938) extremities (*p* = 0.522). On day 1 of NMES, only 64.4% of stimulations led to a contractile response with a significant difference between upper (100%) and lower (41.7%) extremities (*p* = 0.001). This difference could consistently be observed until day 7 (Fig. 1a + b). Nevertheless, a significant correlation for contractile response between upper and lower extremities was observed (*k* = 0.687, *r*^2^ = 0.472, *p* = 0.001). Contractile response declined throughout the 7-day observation period from 64.4 to 25.0% overall. We similarly observed a decrease in contractile response for upper (100.0 to 58.3%) and lower (41.7 to 12.5%) extremities (Fig. 1a + b) (Additional file 1: Table S2b).

Furthermore, the results showed that the proximal muscle groups overall are less likely to respond to NMES than the distal muscle groups (median [IQR] proximal 40.7 [22.5/60.4] vs. distal 75.0 [38.0/91.7] %, *p* < 0.001). This finding is evident in both upper (median [IQR] proximal 73.2 [42.1/88.7] vs. distal 79.1 [72.5/94.6] %, *p* = 0.047) as well as the lower extremities (median [IQR] proximal 8.3 [0.0/45.8] vs. distal 52.5 [3.6/87.9] %, *p* = 0.003). The M. vastus lateralis was the least likely to respond to neuromuscular electrical stimulation (Fig. 1c) (Additional file 1: Table S2b).

The threshold of electrical current that led to a contractile response did not significantly change between day 1 and 7 for all muscle groups taken together (median [IQR] day 1, 50.2 [31.3/58.8] mA; day 7, 45.3 [38.0/57.5] mA) as well as for upper (median [IQR] day 1, 47.9 [24.4/60.5] mA; day 7, 44.4 [32.1/57.9] mA) and lower extremities (median [IQR] day 1, 49.3 [41.8/57.3] mA; day 7, 45.9 [37.1/49.8] mA) separately (Fig. 1d + e). Nevertheless, there was a significant difference between the upper and lower extremities observable on days 2, 3, 4, 5 and 6 with a higher electrical current mostly necessary in the lower extremities. The applied electrical current between upper and lower extremities correlated significantly (*k* = 0.710, *r*^2^ = 0.505, *p* = 0.002) (Additional file 1: Table S2b).

At first adequate awakening, all patients presented with significant weakness overall (median [IQR] 3.2 [2.5/3.8] MRC score) as well as for upper (median [IQR] 3.1 [2.4/3.8] MRC score) and lower extremities (median [IQR] 3.0 [2.9/3.8] MRC score) individually (Fig. 1c, f, g). Until discharge, an increase was observable overall (median [IQR] 3.5 [3.0/3.9] MRC score), for upper (median [IQR] 3.8 [2.9/4.0] MRC score) and for lower extremities (median [IQR] 3.8 [3.1/4.0] MRC score), while statistical significance was only reached for the lower extremities (*p* = 0.042) (Fig. 1f, g). Muscle strength between upper and lower extremities at first awakening as well as at discharge showed a significant correlation (first awakening *k* = 0.864, *r*^2^ = 0.746, *p* < 0.001; ICU discharge *k* = 0.751, *r*^2^ = 0.564, *p* < 0.001).

Using the contractile response cut-off value, 8 patients were classified as responders (> 50% contractile response during the first 7 days) and 13 were classified as non-responders (≤ 50% contractile response during the first 7 days). Univariate analysis revealed a significantly higher SOFA score in non-responders at admission to the ICU. Baseline characteristics were otherwise balanced including norepinephrine treatment (Table 2).

**Table 2:** Univariate analysis

		Responder	Non-responder	*p* value
Patients/stimulations (*n*)		8/702	13/1122	
Sex (m/f)		7/87.5% / 1/12.5%	9/69.2% / 4/30.8%	0.340
Age (years)		56.0 [36.5/71.0]	53.0 [47.0/70.0]	0.645
Weight (kg)		80.0 [70.0/92.5]	92.0 [75.0/109.0]	0.301
Height (m)		1.80 [1.77/1.83]	1.76 [1.70/1.80]	0.500
BMI (kg/m^2^)		26.5 [22.6/29.0]	27.8 [25.5/33.6]	0.210
Diagnosis responsible for ICU admission	ARDS	2/25.0%	6/46.2%	0.118
	Sepsis	0/0.0%	3/23.1%	
	Multiple trauma	4/50.0%	3/23.1%	
	Neurologic	2/25.0%	0/0.0%	
	Miscellaneous	0/0.0%	1/7.7%	
SOFA at ICU admission		12.0 [9.5/13.5]	14 [12.0/16.0]	*0.030*
APACHE II at ICU admission		24.0 [17.0/27.0]	25.0 [23.0/29.0]	0.414
SAPS2 at ICU admission		43.0 [33.0/61.5]	61.0 [57.0/66.0]	0.089
GCS at ICU admission		5.5 [3.0/7.5]	3.0 [3.0/6.0]	0.456
Time until first awakening (days)		12.0 [7.5/15.5]	20.5 [10.0/42.0]	0.287
ICU length of stay (days)		28.0 [19.0/36.0]	39.0 [25.0/49.0]	0.185
Percent of days with RASS > − 3 during ICU stay		50.2 [26.9/94.6]	71.4 [50.0/79.2]	0.750
Noradrenalin (μg/kg min)		0.08 [0.03/0.10]	0.07 [0.06/0.11]	0.414
Time requiring noradrenalin (days)		12.0 [3.5/15.5]	12.0 [11.0/25.0]	0.595
Survivors/non-survivors		7/87.5% / 1/12.5%	11/84.6% / 2/15.4%	0.854
Non-excitable muscle membrane/excitable muscle membrane		2/33.3% / 4/66.7%	5/62.5% / 3/37.5%	0.280
Start of NMES treatment after ICU admission (days)		3.0 (2.0/6.0)	4.0 (2.0/6.0)	0.750

Values for metric variables are presented as median and interquartile range and for categorical variables as count and percentages. Mann-Whitney *U* or chi-square test were used to calculate statistical significance. The statistically significant *p*-value (*p* < 0.05) is italicised to highlight it. BMI = Body Mass Index; SOFA = Sepsis-related Organ Failure Assessment; ICU = intensive care unit; APACHE II = Acute Physiology and Chronic Health Evaluation II; SAPS 2 = Simplified Acute Physiology Score 2; GCS = Glasgow Coma Scale; RASS = Richmond Aggitation Sedation Scale; NMES = Neurmuscular electrical stimulation

**Fig. 2:**
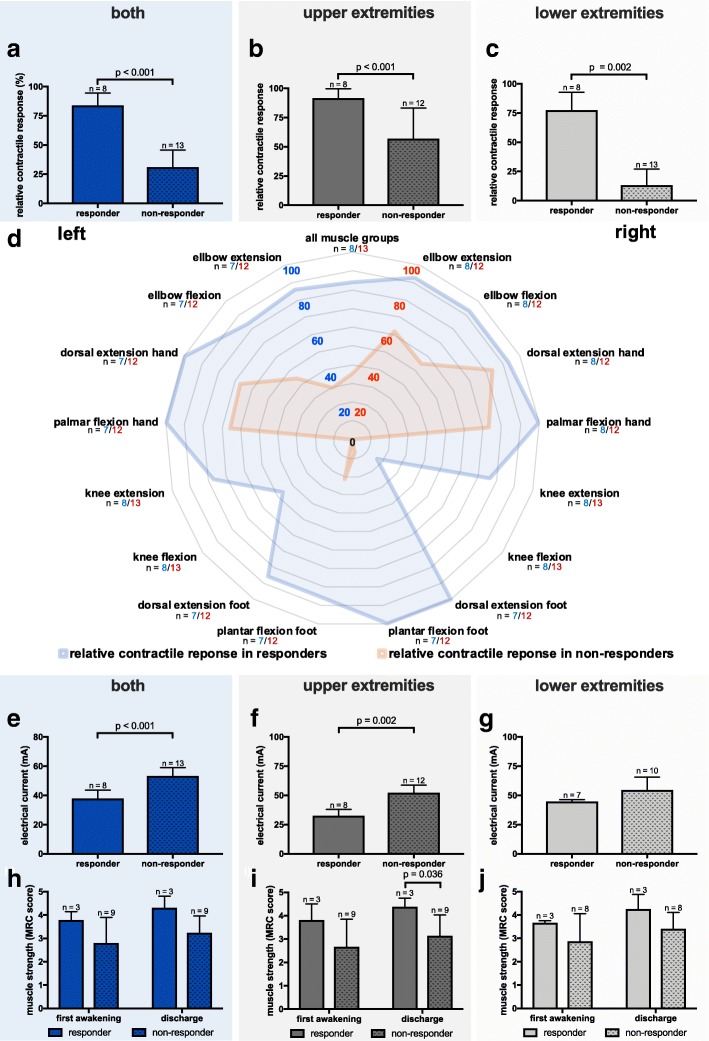
Relative contractile response, electrical current and muscle strength in comparison between responders and non-responders. Relative contractile response is significantly higher in responders as opposed to non-responders during the first 7 days after ICU admission **a** for both extremities, **b** for upper extremities, **c** for lower extremities and **d** for all muscle groups separately. Electrical current required to elicit a contractile response is significantly higher in non-responders as opposed to responders during the first 7 days after ICU admission **e** for both extremities and **f** for upper extremities, while no difference can be observed for **g** lower extremities. Muscle strength measured via MRC scored at ICU discharge shows significantly higher values in responders as opposed non-responders for **i** upper extremities, while not reaching statistical difference for **h** both extremities as well as **j** lower extremities at first adequate awakening as well as ICU discharge and **h** upper extremities at first adequate awakening. All values are shown as median and interquartile range. Statistical significance was calculated via Mann-Whitney *U* test or Wilcoxon signed-rank test as appropriate. MRC = Medical research council

**Fig. 3:**
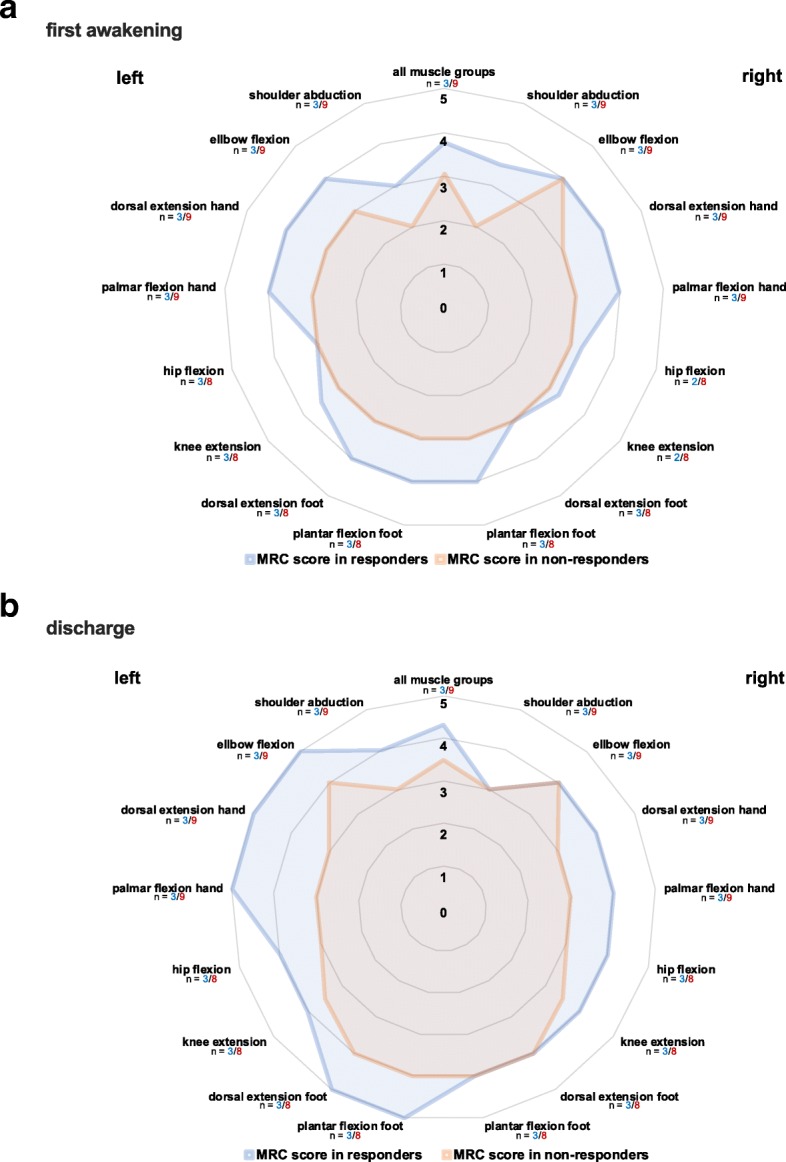
Muscle strength at first adequate awakening and ICU discharge for all muscle groups separately. Muscle strength difference measured via MRC score at **a** first awakening and **b** ICU discharge in responders and non-responders. MRC = Medical research council

When comparing the contractile response from responders to non-responders, we see a significantly greater proportion of stimulations leading to an adequate contractile response in responders (responders vs. non-responders median [IQR] both, 83.7 [73.4/93.5] vs. 35.0 [20.2/44.2] %, *p* < 0.001; upper extremities, 91.1 [86.6/99.1] vs. 67.0 [40.9/74.1] %, *p* < 0.001; lower extremities, 77.7 [62.5/90.2] vs. 7.1 [1.8/23.2] %, *p* = 0.002), indicating that our cut-off value was sufficient in distinguishing between patients that respond well to those who do not (Fig. 2a–d). Interestingly, no decrease in contractile response over time was observed in responders as opposed to non-responders (Additional file 2: Figure S1). When evaluating contractile response for all muscle groups separately, we also observed a consistent, remarkable and significant difference between responders and non-responders. Furthermore, it became evident that except for the dorsal thigh and M. vastus lateralis, the difference in response between upper and lower extremities is only significant for non-responders (Fig. 2d). This difference in contractile response is also reflected by the electrical current that was necessary to elicit a contraction if possible at all, as it was significantly higher in non-responders overall (responders vs. non-responders median [IQR] 38.0 [32.8/42.9] vs. 54.7 [51.3/56.0] mA, *p* < 0.001) and for upper extremities (responders vs. non-responders median [IQR] 32.6 [30.6/37.3] vs. 54.7 [45.4/56.0] mA, *p* < 0.001). Interestingly, no difference in electrical current was observed for lower extremities (responders vs. non-responders median [IQR] 44.6 [31.1/46.1] vs. 54.4 [33.0/58.3] mA, *p* = 0.140) (Fig. 2e–g) (Additional file 3: Table S1). Muscle strength at first awakening showed no statistically significant differences between responders and non-responders in the upper extremities (responders vs. non-responders median [IQR] 3.8 [3.4/4.2] vs. 3.0 [2.0/3.4] MRC score, *p* = 0.145), lower extremities (responders vs. non-responders median [IQR] 3.7 [3.3/3.7] vs. 2.9 [2.4/3.9] MRC score, *p* = 0.630) and overall (responders vs. non-responders median [IQR] 3.8 [3.4/4.0] vs. 3.1 [2.1/3.5] MRC score, *p* = 0.282) (Figs. 2h–j and 3 a). At ICU discharge, a statistically significant difference in muscle strength can be observed with higher muscle strength in the upper extremities of responders (responders vs. non-responders median [IQR] 4.4 [4.1/4.6] vs. 3.3 [2.8/3.8] MRC score, *p* = 0.036), while in lower extremities (responders vs. non-responders median [IQR] 4.3 [3.5/4.6] vs. 3.6 [3.1/4.0] MRC score, *p* = 0.376) and overall (responders vs. non-responders median [IQR] 4.3 [3.8/4.6] vs. 3.5 [2.8/3.9] MRC score, *p* = 0.145) statistical significance was not reached (Fig. 2h–j and 3b).

The proportion of contractile responses to NMES correlated inversely with the applied electrical current overall (*k* = − 0.746, *r*^2^ = 0.556, *p* < 0.001) as well as for upper extremities (*k* = − 0.653, *r*^2^ = 0.427, *p* = 0.002). No correlation between electrical current and contractile response was found for the lower extremities.

The receiver operating characteristics analysis (AUC 0.962; *p* = 0.001) revealed that an electrical current of 50.1 mA elicits a contraction in responders with a sensitivity of 100.0% and a specificity of 84.6%.

**Fig. 4:**
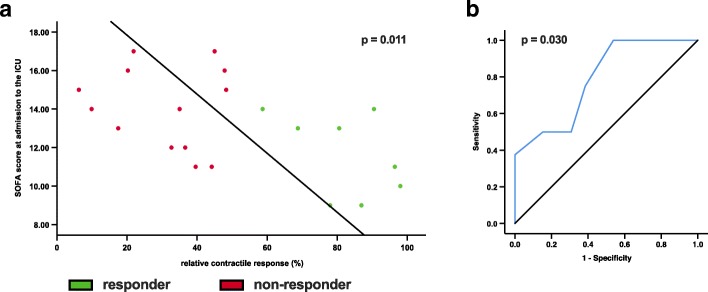
Correlation and ROC curve for contractile response and SOFA. Relative contractile response between days 1 and 7 correlates with **a** SOFA score. **b** ROC curve for SOFA score in patients with adequate contractile response to neuromuscular electrical stimulation. SOFA = Sepsis-related Organ Failure Assessment; ROC = receiver operating characteristics

Contractile response furthermore correlates inversely with the SOFA score at admission (*k* = − 0.544, *r*^2^ = 0.296, *p* = 0.011). A SOFA score ≤ 13.5 has a sensitivity of 75% and a specificity of 61.5% to identify responders (AUC 0.788; *p* = 0.03) (Fig. 4a, b).

Contractile response to NMES presented no statistically significant difference in patients with electrophysiologically diagnosed non-excitable muscle membrane in comparison to patients with an excitable muscle membrane (non-excitable muscle membrane vs. excitable muscle membrane median [IQR] 44.2 [36.1/53.3] vs. 78.0 [42.5/88.7] %, *p* < 0.128).

## Discussion

This retrospective sub-analysis aimed to outline the characteristics of, as well as predictors for, a contractile response to NMES, and also potential clinical benefits resulting from an adequate response to NMES. We were able to show that at the initiation of NMES, only two thirds of stimulations led to a contractile response in our cohort and that this number declined to one third throughout the first 7 days of stimulation, which we think is due to progressing pathophysiological processes. Established mechanisms that would hypothetically contribute to this effect are channelopathy, decreasing metabolic flexibility, advanced muscle atrophy and edema. Interestingly, the upper extremities proved to respond to NMES more frequently than the lower extremities and the distal extremities more frequently than the proximal extremities. If contractile response was present, the required electrical current for a muscle contraction did not change within the first 7 days, while in the lower extremities higher currents were usually required to elicit a contractile response. Patients classified as responders to NMES were shown to have a lower SOFA score, to require a lower electrical current and to have a significantly improved upper extremity muscle strength at discharge. An electrical current of 50.1 mA was sufficient to adequately stimulate responders to NMES, which can be identified by a SOFA score ≤ 13.5.

NMES has been investigated in the context of critical illness and ICU-acquired Weakness for almost a decade without a clear conclusion regarding its effectiveness mainly due to controversial and incoherent study results [13, 14, 17]. As an example, an improvement in muscle strength using NMES was shown by Karatzanos and colleagues [22]. On the other hand, Kho et al. were not able to reproduce these findings [23]. An aspect that is critical when reporting interventional trials is whether the intervention was delivered as planned. Segers et al. first suggested that not every electrical impulse during NMES is converted into a muscle contraction [18]. Nevertheless, most trials on NMES published to date merely report the number or total duration of NMES sessions without mentioning the relative amount of stimulations successfully translated into a muscular contraction, a potentially useful surrogate indicating successful delivery of NMES. A lack of contractile response in the participants could be one cause for the incoherent and contradictory study results in NMES interventional trials throughout the past years. Contractile response was only reported in one outcome-focused interventional trial by Rodriguez and colleagues, who conducted an intraindividual comparison in 16 patients [17]. The muscular response rate of 77% was relatively high compared to our observations as well as to the numbers presented by Segers et al. [17, 18]. Interestingly, they also report an increase in muscle strength at ICU discharge, which is similar to the increased muscle strength we found in the upper extremities of our responder group at ICU discharge [17]. When looking at markers for muscle mass—extremity circumference or muscle thickness via ultrasound—Rodriguez et al. found no effect due to NMES. Interestingly, the dissociation between muscle mass and function has been repeatedly observed in critically ill patients shortly after ICU admission as well as 6 months after ICU discharge [24, 25]. Nevertheless could muscle strength only be assessed via a nonvolitional method based on electrical stimulation of a muscle contraction in the trial investigating the timeframe shortly after ICU admission. The effect of an impaired contractile response has on this form of muscle strength measurement in critically ill patients is unknown [24]. An impaired bioenergetic status has been shown to be of relevance for the development of muscle weakness during the ICU stay and could be a plausible culprit for the difference between muscle mass and muscle function [26]. Future trials are required to elucidate the exact mechanisms.

Our investigation into multiple muscle groups revealed that the M. vastus lateralis has the lowest response rate to NMES and that there is a significant difference between upper and lower extremities in the response to NMES. Based on these facts, we think another factor introducing significant bias into most investigations of NMES is the fact that almost all of them only investigated the lower extremities or solely the quadriceps femoris, which, according to our findings, would carry a high risk of yielding negative study results due to lack of contractile response [13, 14, 23, 27, 28]. A standardised NMES reporting sheet (Additional file 4: Standardised NMES Reporting Sheet) was developed by us in order to improve reporting of NMES protocols and responder rates in future trials and through that enable an adequate comparison of patient cohorts, protocols and muscle groups.

We have shown that a maximum electrical current of 50 mA is sufficient to stimulate all patients that respond adequately to NMES. This number was doubled and almost tripled in some studies, and while we did not observe any harm from NMES with electrical currents up to 70 mA in our cohort, further investigations are needed to rule out any harm from electrical currents above our defined threshold [29].

ICU-acquired weakness has been shown to develop early during critical illness, while the likelihood of developing it increases with the severity of critical illness [15, 16, 30]. In agreement with our results, Segers and colleagues have found that patients with higher disease severity, in their case differentiated by sepsis or no sepsis, are less likely to respond to NMES [18]. Furthermore, we were able to show that distal muscle groups respond better to NMES than proximal muscle groups, which is in accordance with DeJonghe et al., who showed that proximal muscle groups are more severely affected by ICU-acquired weakness than distal muscle groups [31]. Due to the overlap between characteristics of, and risk factors for, ICU-acquired weakness and insufficient response to NMES, it appears likely and plausible that pathophysiological mechanisms involved in the development of ICU-acquired weakness are also responsible for hampering contractile response to NMES [30]. Furthermore, could differences in impedance due to edema or fat mass, a different electrode to muscle ratio or differing adaptive reactions to therapy have an important impact on contractile response [18]. Segers et al. were not able to find an association between electrophysiological characteristics of critical illness myopathy and the lack of contractile response, within our data such an association could not be shown as well but as our analysis is of exploratory nature a large sample size in future trials might yield different results [18]. As such, future investigations specifically dedicated to this question are necessary, in order to further elucidate the connection between electrophysiologic abnormalities and lack of contractile response.

ICU-acquired weakness is a clinical syndrome defined by an average MRC score < 4 [32]. Multiple pathophysiological mechanisms, e.g. muscle wasting, bioenergetic failure and membrane inexcitability, have been described in the context of ICU-acquired weakness [15, 16, 21, 33]. We therefore hypothesise that depending on the aetiology, different pathophysiological mechanisms could be predominantly responsible for the development of ICU-acquired weakness and also be the reason patients respond differently to interventions such as NMES. Future studies should further evaluate these underlying mechanisms in the development of ICU-acquired weakness in order to more closely define different types of ICU-acquired weakness and possibly identify sub-cohorts that respond to and consequently benefit from the different interventions.

Our pilot analysis is limited by the number of patients as multivariate analysis to investigate independency of association between, for example, SOFA score and responder status could not be performed. Furthermore, as our ICU admissions were not planned, we were not able to obtain pre-admission muscle strength values. Our muscle strength values at first awakening will therefore already be affected by the intervention, and correction for potential baseline differences is not possible. We did not included patient with pre-existing neuromuscular disease in order to keep bias due to missing pre-admission values to a minimum. Since our protocol did not include an electrical current above 70 mA, we are not able to reach any conclusion—neither beneficial nor harmful effects—regarding NMES with higher electrical currents. We see that 50.1 mA is a good cut-off to differentiate responders and non-responders on this basis we do not believe increasing electrical current further will increase responder rates.

## Conclusion

Different muscle groups as well as different patients show a varying response to NMES, which appears to be dependent on multiple factors that are linked to the respective pathomechanisms behind the development of the ICU-acquired weakness. Since we observed a varying degree of clinical improvement when comparing patients that responded adequately to NMES to those who did not, it is strongly suggested that response rates to NMES should be reported in future trials (Additional file 4: Standardised NMES Reporting Sheet) and evaluated in relation to clinical outcomes. The described difference in response rates to NMES may explain the controversial findings of previous trials and improve the quality of evidence in future critical care NMES trials.

## Additional files

Additional file 1: **Table S2.** **a** Neuromuscular electrical stimulation reporting: protocol specifications. Specifications of the developed NMES protocol. **b** Neuromuscular electrical stimulation reporting: stimulation and contraction specifications. Specification of the applied NMES. Electrical current shows the median electrical current applied to patients with a positive contractile response per muscle group and day. Contractile response shows the percentage of patients with a positive contractile response per muscle group and day. (XLSX 19 kb)

Additional file 2: **Figure S1.** Contractile response dynamics between day 1 and day 7 in responders and non-responders. (PDF 30 kb)

Additional file 3: **Table S1.** Electrical current. Electrical current necessary to elicit a contractile response for all muscle groups separately for all patients as well as responders and non-responders. (PDF 266 kb)

Additional file 4: Standardised NMES Reporting Sheet. Standardised NMES reporting sheet for NMES protocol and stimulation specifications. (XLSX 17 kb)

## Data Availability

All collected and analysed data can be made available by the corresponding author upon reasonable request.

## Abbreviations

APACHE II: Acute physiology and chronic health evaluation II
AUC: Area under the curve
BMI: Body Mass Index
GCS: Glasgow Coma Scale
ICU: Intensive care unit
IQR: Interquartile range
MRC: Medical research council
NMES: Neuromuscular electrical stimulation
RASS: Richmond Aggitation Sedation Scale
ROC: Receiver operating characteristics
SAPS2: Simplified Acute Physiology Score 2
SOFA: Sepsis-related Organ Failure Assessment
