# Supplementary figures and images for: Differential contractile response of critically ill patients to neuromuscular electrical stimulation
Source: Crit Care. 2019 Sep 10;23:308. doi: 10.1186/s13054-019-2540-4 (PMC6737711; doi:10.1186/s13054-019-2540-4)

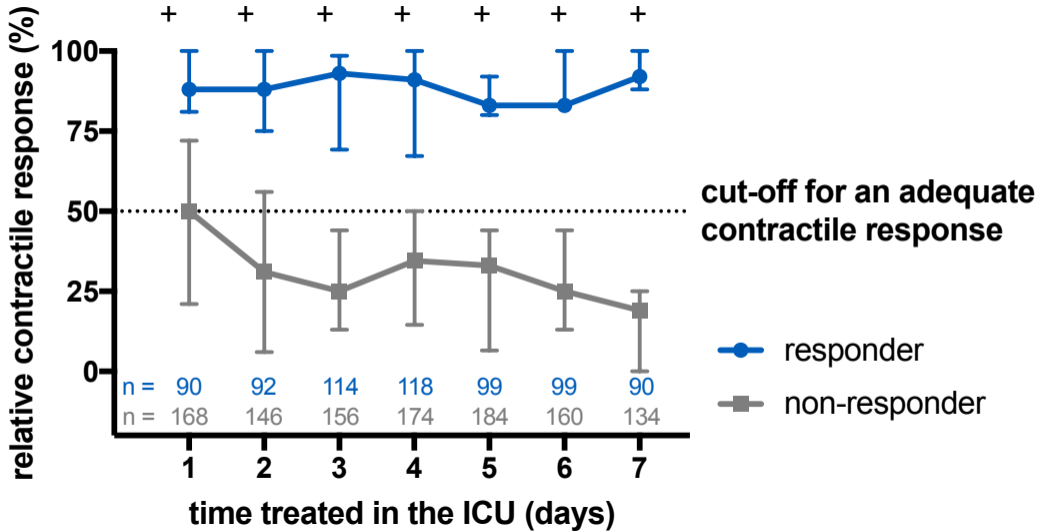

Supplement: Supplementary file 2 — Figure S1. Contractile response dynamics between day 1 and day 7 in responders and non-responders. (PDF 30 kb) [file 13054_2019_2540_MOESM2_ESM.pdf]
