# Supplementary material for: Differential contractile response of critically ill patients to neuromuscular electrical stimulation
Source: Crit Care. 2019 Sep 10;23:308. doi: 10.1186/s13054-019-2540-4 (PMC6737711; doi:10.1186/s13054-019-2540-4)
Supplement: Supplementary file 3 — Table S1. Electrical current. Electrical current necessary to elicit a contractile response for all muscle groups separately for all patients as well as responders and non-responders. (PDF 266 kb) [file 13054_2019_2540_MOESM3_ESM.pdf]

Table S1. Electrical current necessary for a contractile responsen

	All patients	Responders	non-Responders	p-value
M. biceps brachii right (mV)	83.3 [35.7/100.0]	92.9 [83.3/100.0]	54.8 [22.5/81.7]	0.005
M. biceps brachii left (mV)	83.3 [20.0/92.9]	83.3 [83.3/100.0]	57.1 [20.0/84.5]	0.167
M. triceps brachii right (mV)	81.7 [53.6/92.9]	92.9 [84.5/100.0]	61.9 [23.8/80.0]	0.001
M. triceps brachii left (mV)	83.3 [16.7/85.7]	85.7 [84.5/100.0]	29.8 [14.3/79.2]	0.004
Wrist extensors right (mV)	84.5 [75.7/100.0]	92.9 [83.3/100.0]	83.3 [57.1/92.9]	0.208
Wrist extensors left (mV)	75.0 [63.3/85.7]	100.0 [84.5/100.0]	66.7 [39.3/77.5]	0.004
Wrist flexors right (mV)	81.7 [71.4/100.0]	100.0 [91.7/100.0]	73.2 [40.0/90.0]	0.012
Wrist flexors left (mV)	83.3 [60.0/100.0]	100.0 [91.7/100.0]	65.7 [28.6/90.0]	0.013
M. vastus lateralis right (mV)	0.0 [0.0/66.7]	76.2 [66.7/100.0]	0.0 [0.0/0.0]	< 0.001
M. vastus lateralis left (mV)	0.0 [0.0/66.7]	77.4 [66.7/92.9]	0.0 [0.0/0.0]	< 0.001
Knee flexors right (mV)	0.0 [0.0/16.7]	16.7 [0.0/84.5]	0.0 [0.0/0.0]	0.185
Knee flexors left (mV)	14.3 [0.0/33.3]	46.4 [16.7/65.0]	0.0 [0.0/14.3]	0.013
M. tibialis anterior right (mV)	42.9 [0.0/92.9]	100.0 [92.9/100.0]	0.0 [0.0/41.4]	< 0.001
M. tibialis anterior left (mV)	42.9 [0.0/84.5]	85.7 [75.0/92.9]	0.0 [0.0/35.7]	0.003
M. triceps surae right (mV)	28.6 [0.0/100.0]	100.0 [92.9/100.0]	7.1 [0.0/26.8]	< 0.001
M. triceps surae left (mV)	57.1 [7.1/84.5]	85.7 [83.3/100.0]	21.4 [0.0/66.1]	0.003

Values for metric variables are presented as median and interquartile range and for categorical variables as count and percentages. Mann-Whitney U or Chi-Square Test were used to calculate statistical significance. P-value represents comparison between responders and non responders.
